# Sensory deprivation during early development causes an increased exploratory behavior in a whisker-dependent decision task

**DOI:** 10.1002/brb3.102

**Published:** 2012-11-29

**Authors:** Stylianos Papaioannou, Leeann Brigham, Patrik Krieger

**Affiliations:** Department of Neuroscience, Karolinska Institutet, Stockholm Brain InstituteStockholm, Sweden

**Keywords:** Barrel cortex, development, gap-cross, sensory deprivation, whisker tracking

## Abstract

Stimulation of sensory pathways is important for the normal development of cortical sensory areas, and impairments in the normal development can have long-lasting effect on animal's behavior. In particular, disturbances that occur early in development can cause permanent changes in brain structure and function. The behavioral effect of early sensory deprivation was studied in the mouse whisker system using a protocol to induce a 1-week sensory deprivation immediately after birth. Only two rows of whiskers were spared (C and D rows), and the rest were deprived, to create a situation where an unbalanced sensory input, rather than a complete loss of input, causes a reorganization of the sensory map. Sensory deprivation increased the barrel size ratio of the spared CD rows compared with the deprived AB rows; thus, the map reorganization is likely due, at least in part, to a rewiring of thalamocortical projections. The behavioral effect of such a map reorganization was investigated in the gap-crossing task, where the animals used a whisker that was spared during the sensory deprivation. Animals that had been sensory deprived performed equally well with the control animals in the gap-crossing task, but were more active in exploring the gap area and consequently made more approaches to the gap – approaches that on average were of shorter duration. A restricted sensory deprivation of only some whiskers, although it does not seem to affect the overall performance of the animals, does have an effect on their behavioral strategy on executing the gap-crossing task.

## Introduction

Tactile information transmitted via rodents' whiskers is important for the normal development of the physiology and anatomy of the whisker system (Durham and Woolsey 1984; Fox 1992; Ghoshal et al. 2009) and ultimately also whisker-dependent behaviors (Carvell and Simons 1996; Lee et al. 2009). Removing the whiskers, thus changing the normal flow of whisker-mediated information in the whisker system, has effects on the physiology and anatomy that have been studied in considerable detail (Simons and Land 1987; Kossut 1998; Wallace and Fox 1999; Rema et al. 2003; Feldman and Brecht 2005; Shoykhet et al. 2005; Wallace and Sakmann 2008; Krieger 2009; Wimmer et al. 2010). Brain plasticity can refer to three separate but interrelated processes: normal brain development with age, changes caused by sensory experience, and plasticity in response to injury or disease. The degree to which the brain is plastic in any of these senses changes during the life time and thus the concept of “critical periods” has been introduced to refer to the fact that the brain is “plastic” to different degrees during an animal's lifetime. The earlier during development sensory deprivation occurs, the more likely it is that subcortical structures and the formation of the layer 4 barrel pattern are affected, whereas plasticity later in life is predominately cortical in origin and affecting supra- and infragranular layers (Van der Loos and Woolsey 1973; Durham and Woolsey 1984; Simons and Land 1987; Armstrong-James and Callahan 1991; Fox et al. 1996; Glazewski et al. 1998; Wallace et al. 2001). The behavioral consequence of changes to the whisker system includes defects in texture discrimination and defensive behavior (Barneoud et al. 1991; Carvell and Simons 1996; Harris et al. 1999; Shishelova 2006; Celikel and Sakmann 2007; Lee et al. 2009; Shishelova and Raevskii 2010). One behavioral task that can be used to study whisker-related behavior is the gap-crossing test in which the mouse or rat uses its whiskers to estimate the distance to a target platform and subsequently make a jump–no jump decision over the gap separating the two platforms (Hutson and Masterton 1986; Harris et al. 1999; Jenkinson and Glickstein 2000; Sachdev et al. 2000; Celikel and Sakmann 2007; Voigts et al. 2008). We show that a brief but critical period of sensory deprivation has effects on behavior in a whisker-dependent decision-making task. Our study shows the critical importance of the early formation of thalamocortical circuits and the consequences of their permanent changes in the animals' behavior. Specifically, a notable increase in exploratory behavior is triggered when there is an increased necessity for processing in a barrel cortical area where the corresponding whisker was spared during the deprivation. Designing the behavioral task such that the animals must use a whisker that was spared during the sensory deprivation period we studied the (in many cases most relevant) situation where behavior is driven by a sensory organ that remains intact and not damaged.

## Methods

### Animals

C57BL/J6 mice (Charles-River, Germany) of both genders were used for this study. Animals were housed with littermates and mothers with food and water available ad libitum under constant temperature (21°C) with a 12-h light/dark cycle. All procedures were performed in accordance with ethical permits approved by the local ethical committee.

### Sensory deprivation protocols

Previous research suggests that sensory deprivation during the first few postnatal days can induce plasticity in layer 4, while deprivation beginning at P4 or later primarily affects layers 2/3 (Fox 1992). To examine the behavioral consequences of inducing plasticity mainly in layer 4, animals were sensory deprived from P0 to P6. This group will be referred to as the P0 group. At P0 animals (males and females) in each litter were randomly divided into deprived or control groups (P0, *n* = 16; control, *n* = 15). In deprived animals, all whiskers except the C and D rows were plucked unilaterally (right side only) by applying steady tension to the base of the whiskers with forceps under a dissecting microscope. A heating lamp was used to maintain body temperature in animals until fur grew in. P0 animals were plucked once daily. During this period, all animals were handled, including nondeprived littermate controls. Following deprivation, animals were tested beginning at P31–33 on the whisker-dependent gap-crossing task to assess the functional consequences of plasticity. The CD-pairing protocol used for trimming in the P0 group means that the C and D rows on the right side of the face were spared, and all other whiskers were removed. All whiskers were spared on the left side. Thus, during the trimming period, the barrel area (in the left hemisphere) corresponding to the trimmed whisker will not be stimulated by its normal principal whisker.

### Behavioral testing apparatus

The gap-crossing task was performed on two custom-built transparent Plexiglas platforms, one fixed and one movable for manual adjustment of the gap distance between platforms. The apparatus was built essentially as described in Celikel and Sakmann (2007), with two individually moveable identical platforms made of transparent Plexiglas (width = 0.5 cm). The platforms (75 × 220 mm, width × length) were elevated 25 cm off the surface and surrounded on three sides with 20-cm-high walls (Fig. 2). The two platforms were placed end-to-end, facing each other. Each platform is equipped with two motion sensors (MS) to monitor animal movements on the platform and to calculate off-line variables of decision making during the gap-crossing task. Additionally a high-resolution infrared video camera (PIKE 032B, Allied Vision Technologies GmbH, Stadtroda, Germany) fixed above the gap was recording whisker activity during attempts to cross. The platform in the field of view of the camera was called “target platform,” and the platform on the other side of the gap was called “home platform” (Fig. 2). “Target” and “home” are not used to denote a preferred direction of crossings. As the camera is placed over the target platform, data on whisker kinematics (Fig. 5 and Table 1) and nose position (Fig. 4) are only collected when animals are approaching the gap from the home platform. An IR-backlight (Microscan, Renton, WA) positioned below the gap provided necessary contrast for tracking animal and whisker motion. A liquid-cooling block was placed underneath the IR backlight to ensure that a constant temperature was maintained. Extraneous noise was masked with white noise (∼75 dB).

**Table 1:** Whisker kinematics data when the animal is ≤13 mm from the platform

	Mean amplitude (°)	Mean duration (msec)
Control (*N* = 222)		
Protractions	23.15 ± 11.4	11.57 ± 3.285
Retractions	21.84 ± 11.47	8.077 ± 2.397
P0 (*N* = 219)		
Protractions	23.17 ± 10.11	10.94 ± 2.807
Retractions	21.51 ± 10.60	7.986 ± 2.765

### Behavioral training protocol

Two days prior to testing, animals were habituated to the experimenter and apparatus. Each day of habituation consisted of two 5-min sessions of handling, during which the experimenter was interacting with the animals extensively by allowing them to explore his or her hands and by picking them up. Habituation also included 20 min inside the apparatus with the platforms pushed together so that the animals can cross between the platforms without a gap between them. On the first day, the animal was placed inside the apparatus with white noise and the lights on; on the second day, lights were turned off. After the second habituation session, all whiskers except the right C2 were removed to facilitate whisker tracking. The removed whiskers were trimmed with scissors to fur-level or plucked as needed throughout testing. This was done after the test session to avoid stress during the task.

Testing consisted of one session per day for seven consecutive days. Each session lasted 20 min. Animals were placed inside the apparatus with background white noise and in complete darkness. They were allowed to freely explore and cross the gap spontaneously. The gap distance was changed in increments of 0.5 cm after each successful cross according to a pseudorandom protocol that weighted larger distances toward the end of the session. The protocol was divided into five blocks. Within each block, four distances were selected randomly from a predetermined range unique to the block: block 1 = 3–4.5 cm, block 2 = 3.5–5.5 cm, block 3 = 4–6.5 cm, block 4 = 4.5–7 cm, and block 5 = 5–7 cm. This pseudorandom protocol allowed mice to work up to the greater distances while maintaining a degree of unpredictability. Different sets of numbers were generated for each mouse and each session. After each session, the animal was placed back in its home cage and the test apparatus was cleaned with 70% ethanol.

Over the course of the experiment, some animals (control, *n* = 5; P0 group, *n* = 3) lost the spared C2 whisker. Only test sessions prior to whisker loss were included in the analysis. Following the final session, catch trials were performed to ensure that gap crosses were based on sensory input from the whiskers. During these sessions, four trials with distances generated by the pseudorandom protocol were followed by a trial at 8 cm, a distance unreachable with the whiskers. Approximately 25% of animals were randomly selected to participate in catch trials. Of those tested, no animals attempted to cross at 8 cm.

### Analysis of locomotor behavior

The movement of the mouse within the behavioral apparatus was monitored with infrared MS (Fig. 2). The ON and OFF time of the beam breaks from each MS were analyzed in MATLAB using custom-written routines to quantify the temporal dynamics of sensory exploration. Variables that were quantified: number of attempts, duration of the last attempt, and duration of all attempts in a session.

An “attempt” is defined as an event where the animal activates (by breaking the beam) the MS close to the gap (MS2 or MS3 in Fig. 2) and a “successful attempt” is an event where the animal actually crosses over the gap to reach the other platform. The duration of a successful attempt (“duration of last attempt”) is from activation of the MS close to the gap on one side until the activation of the corresponding sensor on the other platform. “Duration of all attempts” includes the duration of the last attempt but also the duration during which MS2 or MS3 was activated but without the animal eventually crossing (thus the duration from MS2-ON until MS2-OFF and MS3-ON until MS3-OFF). In essence, these parameters will quantify how often and for how long time the animal explores the gap.

### Analysis of whisker kinematics

The movement of the whisker was tracked and quantified essentially as previously described (Voigts et al. 2008). The area of the gap between the two platforms was monitored by a high-resolution infrared video camera (Allied Vision Technologies, PIKE 032B) with sampling frequency at 314 Hz at 640 × 300 pixels with resolution of 9.7 pixels/mm.

Tracking of the mouse position and whisker was done off-line on the recorded video sequences as described in Voigts et al. (2008). The algorithm is fully automatized and unsupervised and is implemented in the following steps: The first 50 frames, where there was no mouse detected, were used as an average for background subtraction and normalization of the brightness level. Next, the target platform and the animals nose were detected by simple averaging and thresholding in the *x*-direction. Whiskers were tracked initially as vector fields of polar representation of similarity index extracted by anisotropy functions (i.e., finding the direction of invariance due to blurring and shifting). In a later stage, these paths were integrated and spline interpolated to spatially contiguous representations of whiskers.

Time series of whisking angle were extracted by computing the angle of the whisker's fifth pixel from the base across frames. The angle was calculated in reference to the mean position of the tracked pixel for every sequence. The periods (peak to peak) of this oscillatory signal represented whisking cycles. Whisking cycles were divided into pro- and retraction based on the position of set points (points with zero angular velocity). Whisking amplitude was defined as the angular excursion of the whisker between two set points, respectively, protraction and retraction amplitude. Analysis of frequencies was done by using windowed Fast Fourier Transform of the zero padded time series of whisking angles.

### Analysis of nose position to determine spatiotemporal profile

The spatiotemporal profile describes the probability that at a given time the animal is in a certain part of the gap space. It was calculated as follows. For all tracked frames, the position (*x* and *y* coordinates) of the tracked nose was determined and stored in a 640 × 300 matrix representing the area monitored. The matrix element which corresponds to the nose position was assigned a value of 1 while all other elements were zero. For a sequence of *n* tracked frames, the spatiotemporal profile was created by element-wise addition of all *n* matrices and the resulting sum matrix was normalized to the number of tracked frames *n*. For visualization purposes, the sum matrix was smoothed by convolving with a 5 × 5 pixel matrix. For quantification of the probability, data were collapsed to one-dimensional (1D) by averaging the sum matrix along the *x*-axis.

### Histology

Anatomical changes in barrel formation were also assessed by staining the barrel cortex for cytochrome oxidase. Following behavioral experiments, animals were given a lethal dose of isoflurane by inhalation and perfused transcardially with 20 mL 4% paraformaldehyde or formalin. Brains were removed and postfixed overnight at 4°C. The barrels size was measured from flattened sections cut 100 μm thick. Measurements were made manually with Neurolucida (MicroBrightField Bioscience, VT) from bright-field images.

### Statistics

For each animal, the ratio was calculated as the sum of arcs one to four ([C1 + C2 + C3 + C4 + D1 + D2 + D3 + D4]/[B1 + B2 + B3 + B4 + A1 + A2 + A3 + A4]). As barrel size depends on the barrel arc identity, this later factor appears as a covariate in the barrel size data, which contributes significantly to the sample variance (Airey et al. 2005). Finding the linear relationship between arc identity and barrel size using simple linear regression, we adjusted (normalized) our data by correcting for this effect. The “*n*” for the ratio measurements is thus number of animals × 4 (four barrel arcs). Statistical tests were performed on the adjusted data set. Statistical analysis was done with GraphPad Prism 4 and MATLAB. Box-Cox Power transformation was used to make the data normally distributed, and from this distribution, outliers were defined as ±2 standard deviations. Unpaired two-tailed *t*-test and Kolmogorov–Smirnov test were used to determine statistical significance. Results are presented as mean ± SEM, unless stated otherwise.

## Results

### Effect of sensory deprivation on anatomical staining of layer 4 in barrel cortex

**Figure 1:**
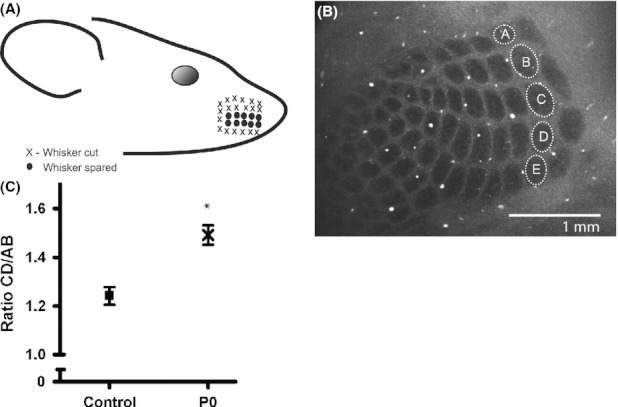
Sensory deprivation causes structural changes in the barrel size. (A) In the sensory deprivation protocol used, the C- and D-row whiskers were spared during different periods of development. (B) Barrels at the level of layer 4 were stained with cytochrome oxidase. The barrels A1–A4, B1–B4, C1–C4, and D1–D4 were traced to calculate barrel area. The dotted circles show schematically the tracing of A1, B1, C1, and D1. Scale bar 1 mm. (C) The ratio of the total adjusted area of the spared (C+D rows)/deprived (A+B rows) in the left hemisphere in control and P0 animals. The ratio was larger for the P0 group compared with control (*P < 0.05).

To analyze whether the sensory deprivation protocol (Fig. 1A) induced structural changes in the somatosensory barrel cortex, we made histological staining to measure barrel size at the level of layer 4. Cytochrome-oxidase staining (Wong-Riley and Welt 1980; Land and Simons 1985) can be used to visualize the size of the barrel columns at the level of layer 4. This metabolic staining overlaps with staining using Vglut-2 (Louderback et al. 2006) to stain for thalamocortical synapses. The size of the barrel column using CO-staining can thus be used to indicate changes in the neural circuitry that can occur as a result of sensory deprivation or deafferentation (Fox 1992; Schlaggar et al. 1993; Lee et al. 2009). To quantify changes in the barrel field size, the area of each individual barrel (A1–A4, B1–B4, C1–C4, D1–D4) was measured (Fig. 1B). The ratio of the total adjusted size of the barrels corresponding to the spared whisker (rows C and D) and the deprived whiskers (rows A and B) within the sensory deprived left hemisphere ([C+D]/[A+B]) was calculated (Fig. 1C). A selective sensory deprivation of only some whiskers during the first postnatal week could decrease the size of the barrel deprived of sensory input similar to that observed by deafferentation (Schlaggar et al. 1993), and the spared/deprived whisker ratio would thus increase because of the decrease in the size of the deprived A- and B-row barrels. The ratio (Fig. 1C) was indeed the higher for the P0 group compared with the control (P0: 1.49 ± 0.04, *n* = 48; control: 1.24 ± 0.04, *n* = 20; mean ± SEM unpaired *t*-test, *P* = 0.0003). These anatomical data indicate that sensory deprivation starting at P0 has effects on the somatosensory barrel circuitry.

### Altered sensory experience, during different periods of postnatal development, does not affect the maximum gap-distance achieved

The gap-crossing task was used to study how decision making based on tactile information from the whiskers is affected by sensory deprivation during the first postnatal week of development, a period critical for the formation of thalamocortical connections. In the “P0 group” only the C- and D-row whiskers were spared (Fig. 1A) between postnatal days 0 and 6 (P0–P6). All whiskers were then left intact from P7 until 2 days before testing (P29–P32), at which time, all whiskers, except the C2 whisker on the right side, were trimmed (cut or plucked). In the littermate controls, all whiskers were left intact until 2 days before testing (P29–P32). Both groups were thus tested with only the C2 whisker on the right-side intact.

**Figure 2:**
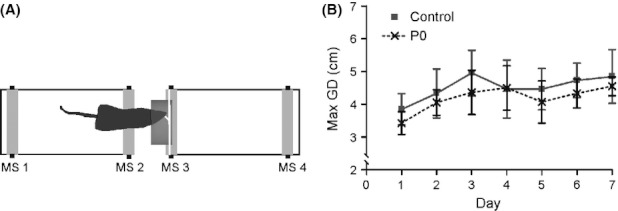
Sensory deprivation did not affect the performance in the gap-crossing task. (A) In the gap-crossing task, the animal is placed on a platform (home platform) and uses its whiskers to judge the distance to the other platform (target platform). Each platform is surrounded by high walls so the only exit is toward the gap separating the platforms. Motions sensors (MS) are used to track the position of the animal on the platform and used to measure how often the animal approaches the gap and how long time it spends exploring the gap. (Not drawn to scale.) (B) The average maximum distance achieved during the 7-day test period was similar in the control and P0 group. On each day only animals that made at least one crossing were included.

The gap-crossing task is performed in complete darkness so that the animals can only rely on tactile information to locate a target platform across a gap (Fig. 2A). Animals from the different groups (control and P0) were tested over a 7-day period with the gap-cross distances increasing over time within each session as determined by a pseudorandom protocol (see Methods). There was no consistent difference between the groups in the average maximum gap-distance achieved during the 7-day testing period (*P* > 0.05, unpaired *t*-test; Fig. 2B). In both groups, the average number of successful attempts was 6 during days 3–7. An average number of 6 successful attempts means, with the training protocol used, that on average the animals were exposed to a maximum gap distance of 5.5 cm. The relative number of animals that made at least one successful jump over the gap increased over time in both groups (chi-square test, *P* < 0.05), suggesting that on average animals in both groups were as likely to perform in the task.

In the testing paradigm used, there were thus no detectable differences in the ability of the animals to perform the task (defined as the increased average gap distance crossed with increased number of training sessions) or the average maximum gap distance achieved at the final day of training. Next, we analyzed whether there was a difference in the behavioral strategy and whisker movements between animals in the different groups.

### Different behavioral strategies to solve the gap-crossing task

To investigate the behavioral strategy the animals use to solve the gap-crossing task, we analyzed how many times they approach the gap and the duration that the animals spend exploring the gap. This measure is used to assess how actively the animals explore the gap. The rationale behind these measurements is that the time that the animal spends exploring the gap before crossing reflects the time for the sensory processing necessary to make a decision. The number of attempts made can be both an index of the general locomotor activity (not only related to solving the gap-crossing task), but also more specifically to the animal's behavioral strategy to solve the gap-crossing task.

**Figure 3:**
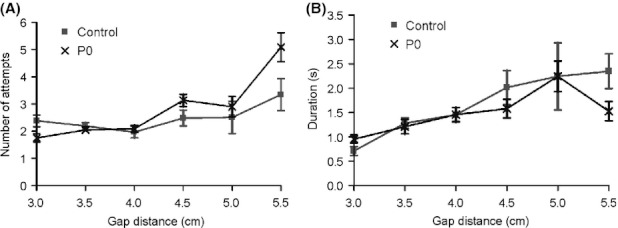
Sensory exploration strategy is affected by sensory deprivation. (A) P0 animals made more attempts to jump over the gap in comparison with control animals. The differences are significant at a gap distance of 5.5 cm, which is the distance where the animals can only rely on their whiskers to contact the target platform. (B) The duration the animal spends exploring the gap is shorter in P0 animals compared with control animals. *P < 0.05. Error bars show mean ± SEM.

The total number of attempts (including both failures and successes) and the total duration of all attempts were similar for both groups up to gap distances of 5 cm (Figs. 3 and S1), but the animal groups clearly deviated at 5.5 cm. At gap distances of 5.5 cm, the P0 group made relatively more attempts (5.1 ± 0.5, *n* [animals] = 12) to cross the gap as compared with the control (3.4 ± 0.6.3, *n* = 10; unpaired *t*-test, *P* = 0.04). The average number of successful attempts on a given day (average range: 3–7) was, however, the same for both groups (unpaired *t*-test, *P* > 0.05). The increased total number of attempts in the P0 group thus means that these animals approach the gap many times without actually jumping. The duration spent exploring the gap was at the longest gap distance (5.5 cm) shorter (unpaired *t*-test, *P* = 0.048) for the P0 group (1.5 ± 0.2, *n* [animals] = 12) compared with control animals (2.3 ± 0.4, *n* = 10).

The similarities between the groups at gap-cross distances up to 5 cm are likely due to the fact that the animals, in addition to using their whiskers to explore the target platform, can also use their nose to touch the platform (Hutson and Masterton 1986). At longer distances, the animals cannot touch with their nose, and thus, the effect of sensory deprivation on whisking-mediated behavior is more prominent.

### The spatiotemporal profile of exploration

**Figure 4:**
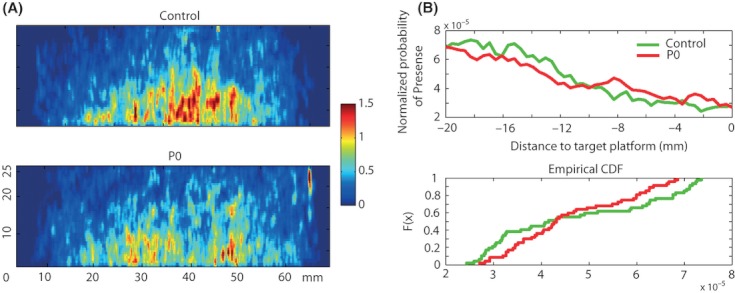
The spatiotemporal profile during exploration of the gap. (A) The animals' spatiotemporal profile was calculated by tracking the nose position in the gap space. For each tracked frame, the *x* and *y* coordinates of the nose were extracted and the profiles were created from all tracked frames. The pseudocolor coding corresponds to the probability of having the nose positioned at the specific area for a given frame. In comparison with control, the spatiotemporal profile for the P0 animals is more homogenously distributed in the gap space. Color scale bar 0–1.5 × 10^−4^.(B) To evaluate the probability of the animal being in a given position (Probability of Presence) along the *y*-axis (which corresponds to distance from the target platform), the two-dimensional data were collapsed in one-dimensional (1D) by averaging along the *x*-axis (upper graph). The cumulative distribution function (F[x]) of the 1D data is shown on the lower graph. The more homogenous spatiotemporal profile for the P0 animals compared with control is evident in the cumulative distribution plot as there is less curvature for the P0 group (a random distribution gives a straight line). A Kolmogorov–Smirnov test showed significant difference between the P0 and control distributions (*P* = 0.0475).

In addition to measuring how many attempts the animal make and how long they stay exploring the gap, we analyzed the position of the nose within the gap space. The spatiotemporal profile during exploration was calculated by tracking the nose position to calculate the probability that the nose is at a given position (Fig. 4). The pseudocolor coding gives the probability of finding the nose position in that point in space. The spatiotemporal profile maps show that in comparison with control animals, the P0 animals spend their time more evenly distributed in the gap space. This is evident by the lack of red colored areas (indicating a high probability) at distance of 10–20 mm from the target platform. The difference can be quantified (Fig. 4B) by comparing the cumulative distributions of the collapsed 1D data showing a significant difference between the P0 and control (Fig. 4B, Kolmogorov–Smirnov test, *P* = 0.0475).

Analyzing the motor behavior thus indicates that as the animal for the decision making must rely more on whisker information, the P0 animals are more active (increased number of attempts; dwell time more homogenously distributed in the gap space). In the next sections, we analyze how these differences in motor behavior are reflected in changes to the acquisition of sensory information using the whiskers.

### Whisker kinematics

One determinant of decision making based on whisker touches is the integration of sensory information collected before reaching a decision (Celikel and Sakmann 2007). The amount of sensory information is determined by the duration the animal spends exploring the gap and the number of contacts with objects that the animal makes with its whiskers. Analyzing the whisker kinematics (whisking cycle amplitude and duration) is thus important for understanding how the mouse has used its whiskers to explore the environment.

**Figure 5:**
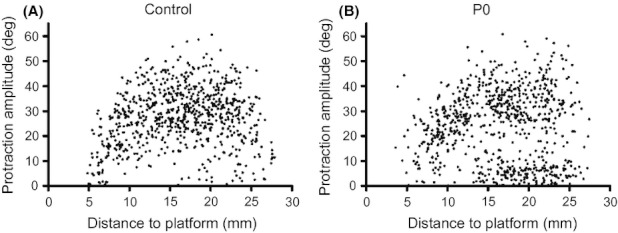
Whisker kinematics as a function of distance from the target platform. (A) For control animals, the whisking amplitude decreases when the animal is within reach of the target platform with its whiskers (at ∼13 mm). (B) In the P0 animals, similarly to that observed for the control animals, the whisking amplitude decreases when the animals touch the target platform, but in addition, the P0 animals are also more actively exploring other areas of the gap; thus, there are more low-amplitude whisks (see text for details) when the animal is exploring the surrounding close to the home platform.

The whisking cycle amplitude and duration was calculated when the animal was at different distances from the target platform (Fig. 5). Furthermore, the whisking cycle was divided (Table 1) into the protraction (whiskers moving forward, away from the body) and the retraction phase (whiskers moving backward, toward the body). Analysis of the whisker kinematics in the control group shows that the amplitude of whisking increases up to a certain distance (∼13 mm) from the target platform at which point the mouse makes contact with the target platform and this triggers a sensory-mediated decrease in whisking amplitude (Fig. 5A). The P0-group animals show a similar change in whisking amplitude as a function of distance to the target platform, but in addition, they have relatively many small-amplitude whisks already before touching the target platform (Fig. 5B). To quantify these whisking cycles (that were not made with the target platform), the proportion of whisking cycles at a distance of approximately 16–28 mm from the target platform with a protraction amplitude less than 15° was calculated (Fig. 5). The proportion of low-amplitude whisking cycles was lower (chi-square test, *P* < 0.0001) for control animals (0.12; 54 of 440 whisking cycles) compared with the P0 group (0.37; 172 of 469 whisking cycles). The control animals thus made, relative to the P0 animals, more low-amplitude whisks (indicative of touches) in the proximity of the target platform. In combination with the analysis of animal position (Fig. 4), this shows that the control animals are more attentive to the target platform compared with the P0-group animals.

A measure of whisker kinematics when the animal actually touches the target platform can be compared between animal groups with study if the touched-induced modulation of the whisking is affected by the sensory deprivation. Comparing the whisker kinematics at distances where the animal can make whisker contacts with the target platform (∼13 mm), there was, however, no difference in the whisker kinematics between the groups (Table 1), indicating that touch-induced whisker modulation is the same as in the control animals. To further examine whisker kinematics, we analyzed the number of contacts the animal makes with its whisker to the target platform in a successful attempt (i.e., an attempt when the animal crossed the gap). The total time during a 200-msec period, before crossing, that the whiskers were in contact with the target platform was similar for both groups (*P* > 0.05, unpaired *t*-test; control 76 ± 44 msec; P0: 84 ± 40 msec).

## Discussion

Using an experimental paradigm to selectively deprive/spare the sensory input to different parts of the somatosensory barrel cortex, we have studied how sensory deprivation, induced during a period that is critical for normal formation of thalamocortical connections, has affected whisker-dependent behavior. We tested the animals' cognitive ability in the gap-crossing task where they had to use sensory information from a spared whisker to judge the distance to a platform and decide to jump or not to jump over the gap to the other platform. We show that sensory deprivation during early postnatal development changes the animals' explorative behavior; that is, they become more active in making more attempts of shorter duration.

### Barrel pattern development and brain wiring

We studied the behavioral effect of sparing only the CD rows from P0 to P6. This manipulation is done during a “critical period” of barrel cortex plasticity (Durham and Woolsey 1984; Fox 1992; Simons and Land 1994). A change in the responsiveness of layer 4 neurons is primarily affected by sensory deprivation protocols applied within the first postnatal week, whereas neurons in layers 2/3 retain their plasticity throughout adolescence (Armstrong-James et al. 1994; Diamond et al. 1994; Glazewski and Fox 1996; Lendvai et al. 2000). The layer 4 plasticity is presumably mainly caused by changes in the thalamocortical innervation of layer 4 (Woolsey and Wann 1976). As the marker for thalamocortical synapses (vGlut2; Fremeau et al. 2001; Kaneko and Fujiyama 2002) is correlated with the metabolic CO-staining, the size of the CO-barrel staining can be interpreted as showing the area innervated by thalamocortical synapses (Louderback et al. 2006). A change in the CO-stained barrel area is shown for the P0 group where the barrel area ratio of spared (C+D row)/deprived (A+B row) increased. A whisker paring protocol similar to the one used in this study has been shown to cause a decrease in the number/density of axonal projections and the spread of activity from a spared to a deprived barrel column (Broser et al. 2008; Wallace and Sakmann 2008). In general, sparing a whisker increases its cortical representation (Simons and Land 1987; Diamond et al. 1993; Glazewski and Fox 1996; Wallace and Fox 1999; Glazewski et al. 2000). In this study, we used a sensory deprivation protocol that caused a reorganization in the relative size of the cortical areas activated by a given whisker, with the aim of studying the behavioral effects of such a change. The changes in the size of the barrel patterns could reflect that the underlying mechanism is an over excitation of an enlarged spared cortical area in combination with a decreased inhibition from the reduced neighboring sensory-deprived cortical areas.

### Behavioral performance in the gap-crossing task

Changes in the normal sensory-driven development of somatosensory barrel cortex during different periods of development are shown in this study to affect the behavioral strategy the young adult animals use to solve a decision-making task. The behavioral strategy necessary to solve a task can be analyzed in terms of how many times it is necessary to try and how long it takes to solve the task. CD pairing (the C- and the D-row whiskers are spared all other whiskers removed) during the first postnatal week results in a behavior where the animals make an increased number of shorter duration approaches to the gap (Fig. 3) they also expanded their exploration area close to the gap (Fig. 4). In the P0 group, there was no evident sign of an impaired whisker kinematics as the touch induced modulation of whisker kinematics (Fig. 5), and the number of whisker contacts made with the target platform did not differ between the control animals and sensory deprived animals. Thus, the observed differences in behavior were more likely due to impaired sensory processing and not due to changes in the sensory input per se.

In rats, removing all whiskers for a short period (P0–P3) caused an increase in the barrel size, made the animals reach shorter maximum gap-cross distances, and caused an increased exploratory activity (Lee et al. 2009). In contrast, in this study the CD-paring (the C- and the D-row whiskers are spared all other whiskers removed) during the first postnatal week decreased the area of barrels where the corresponding whisker had been deprived and did not affect the maximum gap distance achieved. The CD-paired P0 groups did, however, at gap distances where the animal had to rely more on their whiskers, make more attempts compared with control animals which could indicate an increased exploratory activity similar to that seen by depriving all the whiskers (Lee et al. 2009). Noteworthy is, however, that the P0 animals show the increased activity at a distance where the importance of whiskers is higher; thus, it is an increased motor activity that is tactile dependent initiated by increased requirements on sensory processing in the somatosensory cortex. The structural arrangement of the whisker in rows and arcs makes it possible to alter sensory experience in many different ways (Ebner and Armstrong-James 2005; Feldman and Brecht 2005). In general, it appears that the effects of removing all whiskers are quite different from removing only a selected few where neighboring barrel columns receive unequal amounts of sensory input (Diamond et al. 1993; Finnerty et al. 1999; Finnerty and Connors 2000; Ebner and Armstrong-James 2005; Wallace and Sakmann 2008; Krieger 2009). These differences in cellular effects caused by different deprivation protocols are apparently also manifested as differences in behavior.

### Explaining the altered behavior in terms of the underlying neuronal circuits

The difference in species (rat or mouse), deprivation protocol (removing all whiskers or only a selected number of rows), duration of deprivation (days or weeks), and other factors complicate a direct comparison between studies of behavior, anatomy, and physiology, which would be necessary to explain the observed behavioral effects in terms of the underlying mechanisms. Our main assumption is that in the P0 group we have interfered with the normal formation of thalamocortical synapses and the preferential spread of intracortical axons along a row (Keller and Carlson 1999). The abnormal formation of thalamocortical projections could result in inadequate sensory gating which is manifested as hyperactivity and attention deficits (reviewed in Cascio 2010). In the experimental paradigm reported in this study, the abnormal formation of thalamocortical projections is manifested as an increased whisker-mediated motor behavior (increased number of attempts with on average shorter duration; Fig 3). The rewiring of the thalamocortical projections, which is evident from the changes in barrels size (Fig. 1), could thus result in an increased cortical representation of the spared whisker and in addition a decrease in the surround inhibition (Kelly et al. 1999) from sensory deprived neighboring relatively smaller barrels (Fig. 1C), these effects could contribute to an over excitation manifested in an increased behavioral activity.

## Supporting Information

**Figure S1.** Data plotted per animal. Each point at each gap distance is from one animal. The number of attempts for control (A) and P0 (B) animals. The duration of an attempt in control (C) and P0 (D) animals. Error bars show mean ± SEM. Not all animals crossed at all gap distances (Control: *n* = 12; P0: *n* = 15).
