# Supplementary material for: Sensory deprivation during early development causes an increased exploratory behavior in a whisker-dependent decision task
Source: Brain Behav. 2012 Nov 29;3(1):24–34. doi: 10.1002/brb3.102 (PMC3568787; doi:10.1002/brb3.102)
Supplement: Supplementary file 1 [file brb30003-0024-SD1.pdf]

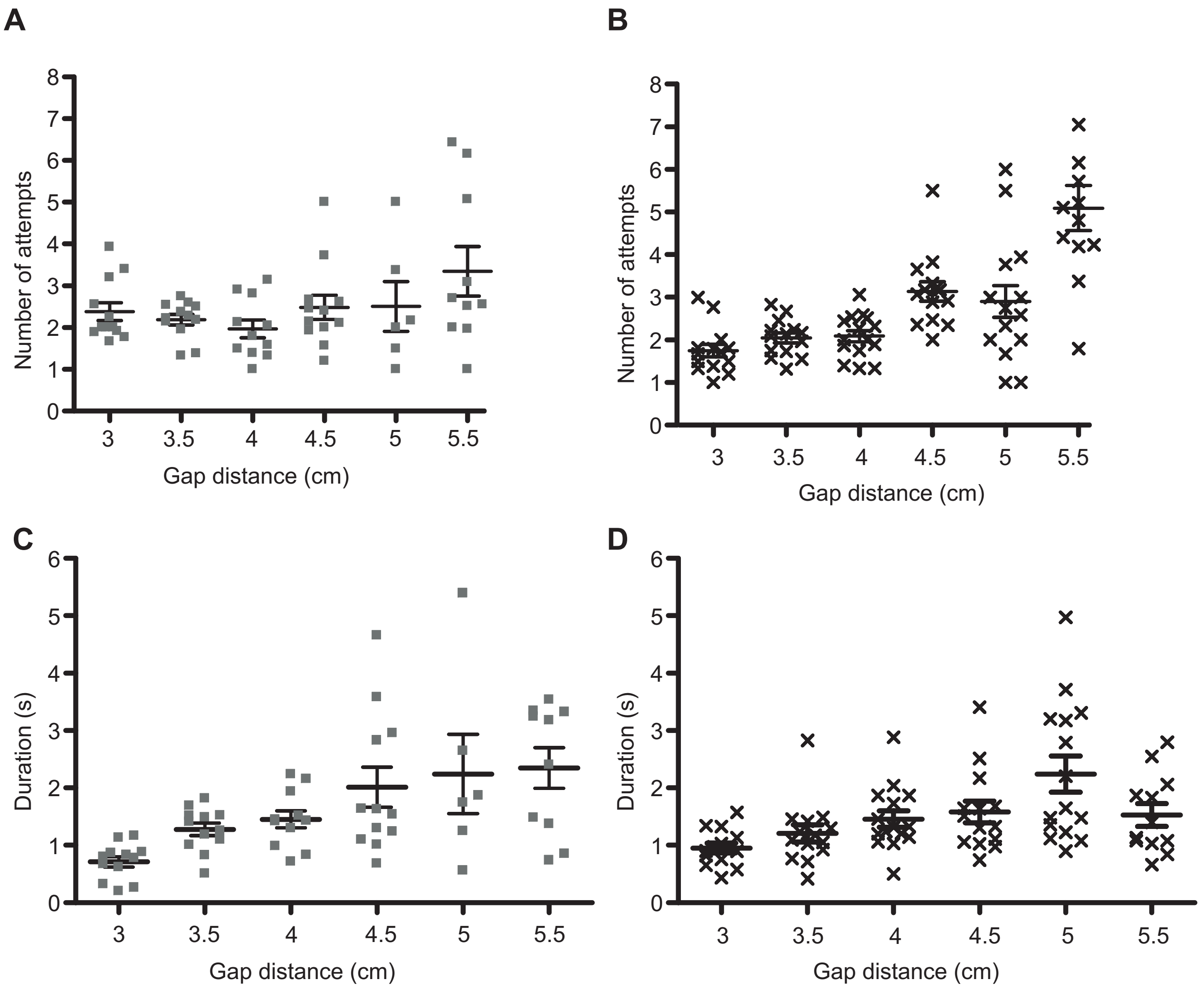

Supplementary Figure 1: Data plotted per animal. Each point at each gap distance is from one animal.Not all animals crossed at all gap distances (Control n=12; P0 n=15) The number of attempts for control (A) and P0 (B) animals. The duration of an attempt in control (C) and P0 (D) animals. Error bars show mean ± SEM
