# Supplementary figures and images for: Sensory deprivation during early development causes an increased exploratory behavior in a whisker-dependent decision task
Source: Brain Behav. 2012 Nov 29;3(1):24–34. doi: 10.1002/brb3.102 (PMC3568787; doi:10.1002/brb3.102)

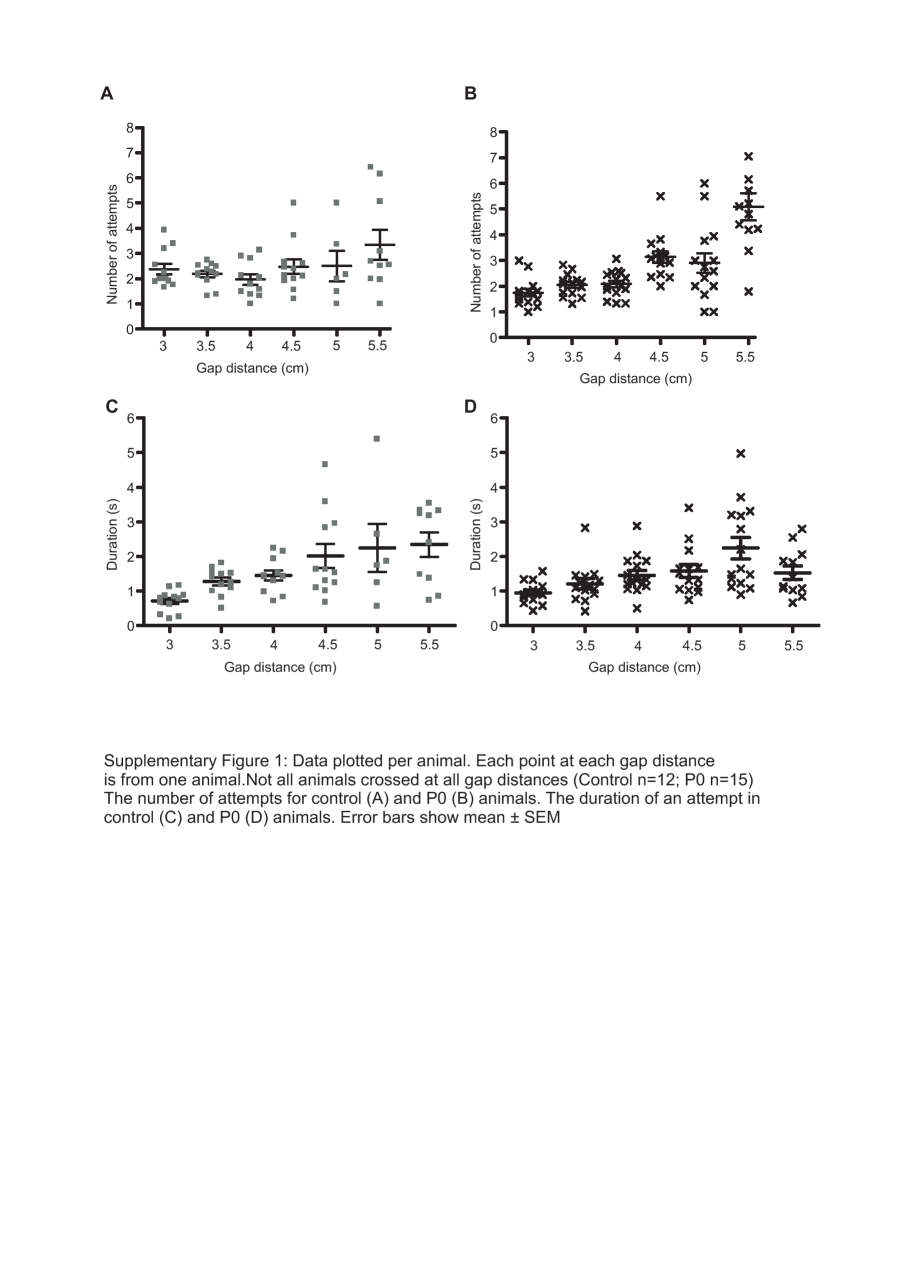

Supplement: Supplementary file 2 [file brb30003-0024-SD2.png]
